# Disease Status–Dependent Drug–Herb Interactions: NASH Lowered the Risk of Hepatotoxicity in Rats Coadministered With Simvastatin and *Gardenia jasminoides* J. Ellis

**DOI:** 10.3389/fphar.2021.622040

**Published:** 2021-04-23

**Authors:** Ziwei Li, Yuanfeng Lyu, Jiajia Zhao, Dan Li, Zhixiu Lin, Kenneth Kin Wah To, Xiaoyu Yan, Zhong Zuo

**Affiliations:** ^1^ School of Pharmacy, The Chinese University of Hong Kong, Shatin, Hong Kong; ^2^ School of Chinese Medicine, The Chinese University of Hong Kong, Shatin, Hong Kong

**Keywords:** simvastatin, gardeniae fructus, nonalcoholic steatohepatitis, hepatotocixity, herb–drug interaction

## Abstract

Concurrent use of simvastatin (SV) and *Gardenia jasminoides* J. Ellis (GJ) was adopted in patients with multi-morbidity, such as stroke rehabilitation patients with NASH. Although hepatotoxicity has been reported in both of them and NASH could alter the pharmacokinetics of drugs/herbs, the interaction between SV and GJ and the related hepatotoxicity remained uninvestigated under neither healthy nor NASH condition. The current study aimed to evaluate the potential hepatotoxicity resulted from the interactions between SV and GJ in both healthy and NASH rats. Both healthy and NASH rats received two-week SV (p. o., 8.66 mg/kg, once daily) and/or GJ (p.o., 325 mg/kg, twice daily). Pharmacokinetic profiles of SV, simvastatin acid (SVA, active metabolite of SV), and geniposide (major component in GJ); hepatic Cyp2c11/Oatp1b2/P-gp expression; and biomarker levels of liver function, lipid levels, and liver histology were compared to demonstrate the interactions in rats. To explore the mechanism of the interaction-mediated hepatotoxicity, hepatic genipin-protein adduct content and iNOS/COX-1/COX-2 expressions from related groups were compared. Moreover, liver histology of healthy/NASH rats at 90 days after discontinuation of two-week GJ in the absence and presence of SV was evaluated to estimate the long-term impact of the interactions. GJ reduced the systemic exposures of SV and SVA by up-regulating the hepatic P-gp expression in healthy but not NASH rats. Meanwhile, SV increased the systemic exposure of geniposide *via* inhibiting the activity of P-gp in both healthy and NASH rats. Although neither SV nor GJ induced hepatotoxicity in healthy rats, their co-treatment elevated serum ALT and AST levels, which may attribute to the aggravated genipin-protein adduct formation, inflammation infiltration, and iNOS/COX-1 expressions in the liver. In NASH rats, SV and/or GJ reduced serum ALT, AST, LDL/vLDL, and TC levels via alleviating hepatic inflammation infiltration and iNOS/COX-1 expressions. Moreover, in comparison to NASH rats, more severe fibrosis was observed in the livers of healthy rats at 90 days after discontinuation of two-week SV and GJ coadministration. Although interactions between SV and GJ induced short-term and long-term liver injuries in healthy rats, NASH condition in rats could lower such risk.

## Introduction

**FIGURE 1:**
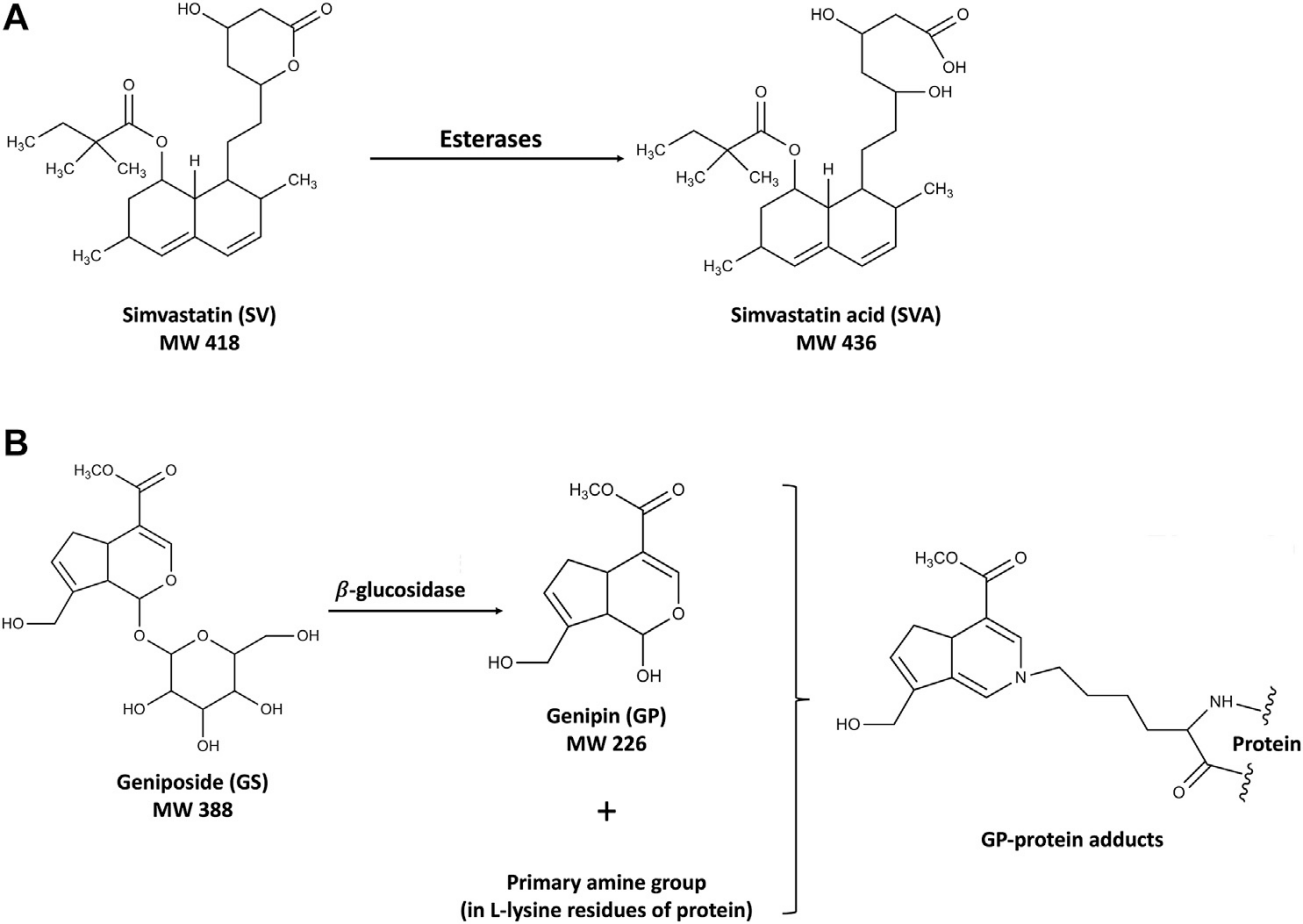
Structures of SV and SVA **(A)** and the mechanism of the hepatotoxicity induced by GS and GP **(B)**.

Simvastatin (SV) is a first-line drug for the primary and secondary prevention of coronary artery disease and one of the most widely prescribed drugs worldwide. *Gardenia jasminoides* J. Ellis (GJ) is a widely used traditional Chinese medicine, and its ripe fruit has been applied in over two hundred traditional Chinese medicinal preparations for various diseases, including brain injuries during stroke rehabilitation, hepatic disorders, and acute jaundice (Zhang et al., 2006; Chen et al., 2015; Chen et al., 2020). In our pilot integrative medicine practice program for fifty stroke rehabilitation patients, elevations of ALT/AST levels were observed in four patients with symptoms related to nonalcoholic steatohepatitis (NASH) who have been using SV together with herbal medicines including GJ. SV is the substrate of CYP3A4/Cyp2c11 (Prueksaritanont et al., 1997; Ishigami et al., 2001) and inhibitor of P-gp (Wang et al., 2001), while its active metabolite simvastatin acid (SVA) is the substrate of both CYP3A4/Cyp2c11 (Prueksaritanont et al., 2003) and transporters such as OATP1B1/Oatp1b2 (Pasanen et al., 2006) and P-gp (Keskitalo et al., 2008). On the other hand, geniposide (GS), the major component of GJ, was found to be the substrate of P-gp in the intestine (Yu et al., 2017), and its active metabolite genipin (GP) has been reported to decrease the expressions and activities of CYP2C19 and CYP3A4, and increase the expression of P-gp (Gao et al., 2014). In the context, coadministration of SV and GJ was expected to result in pharmacokinetic interactions. In addition, such interactions might be further changed under NASH condition, given that OATP1B1/Oatp1b2, CYP3A4/Cyp2c11, and P-gp have been reported to be altered in NASH patients/animals (Donato et al., 2006; Hardwick et al., 2013; Canet and Cherrington, 2014). Moreover, there might exist pharmacodynamic interaction between SV and GJ, leading to potential hepatotoxicity. Despite well tolerance of SV, severe liver injuries resulted from drug–drug interactions of it have been observed in clinics (Ricaurte et al., 2006; Tuteja et al., 2008). It is noteworthy that both GJ and GS have been reported to be hepatotoxic at high dose (Yamano et al., 1990). After oral administration, GS would be hydrolyzed to GP, which could interact with the primary amine groups in the lysine residue of proteins to form blue hepatotoxic GP-protein adducts (Li Y. et al., 2019), as shown in Figure 1. Besides, beneficial effects of both SV and GS on NASH patients and animals have been previously reported (Ma et al., 2011; Athyros et al., 2014), and NASH patients commonly have various comorbidities such as hyperlipidemia and hypertension, which may result in concomitant use of SV and GJ (Higashino et al., 2014).

Herb–drug interaction is a rising public health concern along with the growing prevalence of herbal medicines worldwide (Robinson and Zhang, 2011). Moreover, it is increasingly recognized that the disease status may also participate in the herb/drug–drug interactions. For example, both tocilizumab and sarilumab could alleviate the inflammatory factor IL-6 in patients with rheumatoid arthritis, and therefore reverse the increased IL-6–mediated suppression of CYP3A4 and further reduce the elevated systemic exposures of simvastatin and simvastatin acid in such disease status (Schmitt et al., 2011; Lee et al., 2017). However, the interaction between SV and GJ has never been investigated in neither healthy nor NASH subjects and animals so far. Considering that many patients who have been used SV for long term could be prescribed with GJ and some of them might have NASH, it is of great importance to investigate their interactions in both healthy and NASH conditions. Based on previously reported hepatotoxicities induced by drug–drug interactions of SV and high dose of GJ and its active components GS, it is hypothesized that interactions between SV and GJ might lead to potential liver injury. Such herb–drug interactions might be further influenced under NASH condition due to their altered expressions of various metabolic enzymes and transporters. Therefore, the current study was proposed aiming to investigate the potential pharmacokinetic interactions as well as the short-term and long-term effects of the pharmacodynamic interactions between SV and GJ in both healthy and NASH rats. Furthermore, the mechanisms of such interactions in both healthy and NASH conditions were explored.

## Materials and Methods

### Materials

Simvastatin (>98%), simvastatin sodium salt (>98%), and lovastatin (>98%) were bought from Cayman Chemical (Michigan, United States). Geniposide (>98%), genipin (>98%), and paeoniflorin (>98%) were purchased from Must Inc. (Chengdu, China). *Gardenia jasminoides* J. Ellis concentrated granules were purchased from Purapharm (batch no. A1700325, Hong Kong SAR, China), and the concentrations of GS (10.50%, w/w) and GP (<0.05%, w/w) in it were quantified by HPLC-UV (Supplementary Material). 3-cc Oasis®MCX SPE cartridges were purchased from Waters (Milford, MA, United States). L-lysine (>98%) was purchased from Sigma-Aldrich (St. Louis, MO, United States). Acetate acid, acetonitrile, ethyl ether, dichloromethane, and propylene glycol were of HPLC grade and purchased from RCI Laboscan Ltd. (Bangkok, Thailand). Polyethylene catheter was purchased from Portex Limited (Hythe, England).

Normal chow (rodent diet 50 IF/6F) and high-fat high-cholesterol (HFHC) diet were purchased from Test Diet (St. Louis, MO, United States). Commercial kits of alanine transaminase (ALT, cat. no. ab105134), aspartate transaminase (AST, cat. no. ab105135), low-density lipoprotein/very low-density lipoprotein (LDL/vLDL, cat. no. ab65390), and total cholesterol (TC, cat. no. ab65359) and primary antibodies of P-gp (cat. no. ab170904), Oatp1b2 (cat. no. ab224610), Cyp2c11 (cat. no. ab3571), iNOS (cat. no. ab15323), COX-1 (cat. no. ab109025), and COX-2 (cat. no. ab15191) were purchased from Abcam (Cambridge, MA, United States).

Madin-Darby Canine Kidney II overexpressed with human MDR1 (MDCKII-MDR1) cell line was a kind gift from Prof. Kenneth To. Dulbecco’s modified Eagle’s medium (DMEM), 0.25% trypsin-EDTA, fetal bovine serum (FBS), nonessential amino acids (NEAA), penicillin-streptomycin (10,000 units/mL penicillin G sodium and 10,000 μg/ml streptomycin sulfate), and Hank’s balanced salt solution (pH 7.4, HBSS) were purchased from Gibco BRL (Carlsbad, CA, United States). Rat tail collagen (type I), 3-(4,5-dimethylthiazol-2yl)-2,5-diphenyltetrazolium bromide (MTT), was purchased from Sigma Chemical Co. (St. Louis, MO, United States). Twelve-well Transwell® inserts (12 mm i. d., 0.4 μm pore size, 1.12 cm^2^, polycarbonate filter) and the 12-well culture plates (22.1 mm i. d., 6.9 ml well volume) were purchased from Corning Costar Co. (NY, United States). The 96-well plates were supplied by Thermo Fisher Scientific. The tissue culture flask with filter cap was provided by SPL Life Science (Korea).

### Animal Grouping, Treatment, and Sampling

**FIGURE 2:**
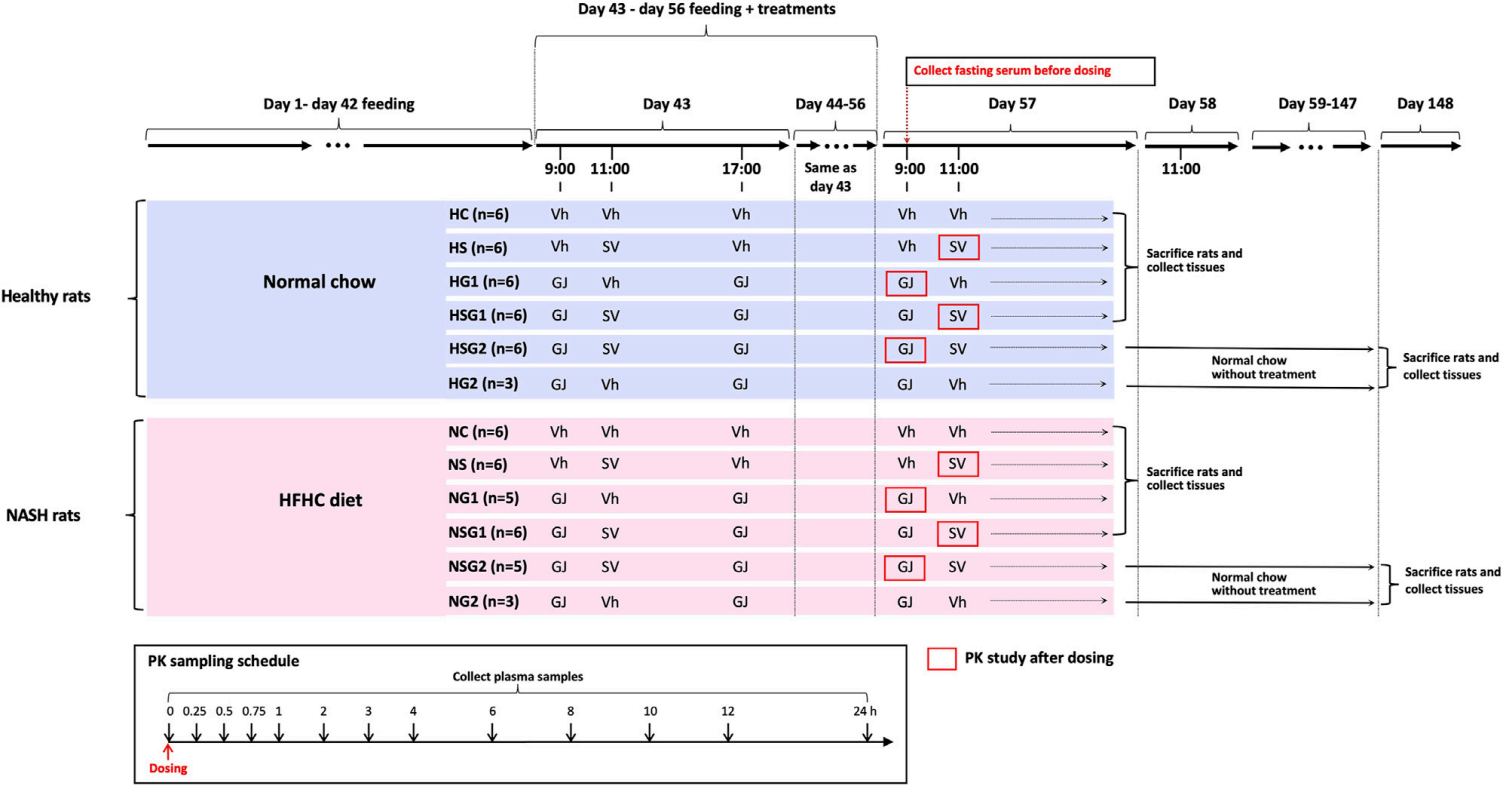
Animal grouping, dosing, and sample collections for the investigations of pharmacokinetic and pharmacodynamic interactions between SV (8.66 mg/kg, p. o.) and GJ (325 mg/kg, p. o.). HFHC, high-fat high-cholesterol; Vh, vehicle. H and N denoted healthy and NASH, respectively. C, S, and G indicated that the rats received vehicle, SV, and GJ, respectively.

Male Sprague-Dawley (SD) rats (six weeks old) were supplied by the Laboratory Animal Services Center at the Chinese University of Hong Kong and kept at ambient temperature (25°C) and relative humidity (50%) with 12 h light/darkness cycle. The animal studies were conducted after the approval of the Animal Ethics Committee of the Chinese University of Hong Kong (Reference number 18-049-MIS). A total of sixty-four male SD rats (six weeks old) were randomly divided into twelve groups as shown in Figure 2. Rats from groups HC, HS, HG1, HSG1, HSG2, and HG2 were fed with normal chow for eight weeks (56 days), while those from groups NC, NS, NG1, NSG1, NSG2, and NG2 received HFHC diet during the same period. In the name of the groups, H and N stand for healthy and NASH, respectively. C, S, and G indicate that the rats received vehicle, SV, and GJ, respectively. HS, HG1, HSG1, HSG2, NS, NG1, NSG1, and NSG2 were used to investigate the pharmacokinetic interactions between SV and GJ in healthy and NASH rats. Meanwhile, HC, HS, HG1, HSG1, NC, NS, NG1, and NSG1 were used to investigate the pharmacodynamic interactions between SV and GJ in healthy and NASH rats after the two-week treatment. Besides, HSG2, HG2, NSG2, and NG2 were used to explore the long-term effect of the two-week coadministration of SV and GJ in healthy and NASH rats.

On day 44, all the twelve groups of rats started receiving different treatments of SV/GJ/vehicle for the following 14 consecutive days (day 43–day 56). SV (1.732 mg/ml) and concentrated GJ granules (65 mg/ml) were suspended in 0.3% sodium carboxymethylcellulose (in double-distilled water) and warm double-distilled water, respectively. The SV suspension, GJ suspension, and their corresponding vehicles were administered to rats at a dose of 5 ml/kg by oral gavage. Rats from groups HC (*n* = 6) and NC (*n* = 6) received vehicles at 9:00, 11:00, and 17:00, while those from groups HS (*n* = 6) and NS (*n* = 6) were treated with SV (8.66 mg/kg) at 11:00 and vehicles at 9:00 and 17:00. GJ (325 mg/kg) were administered to rats from groups HG1 (*n* = 6), HG2 (*n* = 3), NG1 (*n* = 5), and NG2 (*n* = 3) at 9:00 and 17:00 followed by receiving vehicle administered to the four groups of rats at 11:00. Rats from groups HSG1 (*n* = 6), HSG2 (*n* = 6), NSG1 (*n* = 6), and NSG2 (*n* = 5) all received GJ (325 mg/kg) at 9:00 and 17:00 and SV (8.66 mg/kg) at 11:00.

Cannula-insertion surgery was carried out in each of the rats from groups HS, HG1, HSG1, HSG2, NS, NG1, NSG1, and NSG2 on day 56. Briefly, the rat was anesthetized by an intramuscular injection of ketamine (60 mg/kg) and xylazine (6 mg/kg) followed by insertion with a polyethylene catheter (0.50 mm ID, 1.00 mm OD) into their right jugular vein and allowed to recover for one day. On day 58, 200 µL fasting blood (fasted for 12 h with water supplied ad libitum) was withdrawn from the catheter of each rat and placed for 1 h at room temperature followed by centrifugation at 8,000 g for 3 min to obtain fasting serum samples, which were stored at −20°C for the detection of biomarkers. For pharmacokinetics study, at 11:00 on day 58, SV was administered to each rat from groups HS, HSG1, NS, and NSG1 with 200 µL of blood collected from their jugular vein catheter at 0, 0.25, 0.5, 0.75, 1, 2, 3, 4, 6, 8, 10, 12, and 24 h after-dosing into the heparinized tubes that containing 10 µL mixture of 400 mM BNPP and 1 M KF, respectively. As to each rat from groups HG1, HSG2, NG1, and NSG2, after the administration of GJ at 9:00 on day 58, 200 µL of blood was collected from the jugular vein catheter at 0, 0.25, 0.5, 0.75, 1, 2, 3, 4, 6, 8, 10, 12, and 24 h into the heparinized tubes, respectively. The collected blood samples were centrifuged to obtain plasma samples for the determinations of SV, SVA, and/or GS.

After the blood sampling for pharmacokinetic studies, all the rats from groups HC, HS, HG1, HSG1, NC, NS, NG1, and NSG1 were sacrificed via overdose of anesthesia (120 mg/kg ketamine and 15 mg/kg xylazine, i. p.) followed by systemic perfusion with cool saline, and the small intestine and liver were collected and stored at −80°C for further use. Besides, one piece of liver was fixed with 4% paraformaldehyde solution and then embedded in paraffin. In addition, the rats from groups HSG2, HG2, NSG2, and NG2 (*n* = 3 per group) were held for another 90 days with feeding of normal chow and no treatment of any herb/drugs. On day 148, the rats were sacrificed via overdose of anesthesia (120 mg/kg ketamine and 15 mg/kg xylazine, i. p.) followed by systemic perfusion with cool saline, and the livers were collected and stored at −80°C for further use with one piece of which fixed by 4% paraformaldehyde solution and then embedded in paraffin.

### LC-MS/MS Quantification of Simvastatin, Simvastatin Acid, and/or Geniposide in Plasma of Healthy and Nonalcoholic Steatohepatitis Rats

The rat plasma samples collected from groups HS, HSG1, NS, and NSG1 after the last dosing of SV were processed for the quantifications of SV and SVA, following our previously reported method (Li Z. et al., 2019). Briefly, to 100 µL of plasma sample, 50 µL of 100 mM ammonium acetate (pH 4.5) and 10 µL of internal standard (IS, 200 ng/ml lovastatin in acetonitrile) were added followed by addition of 1 ml of mixture of diethyl ether and dichloromethane (3:2, v/v). After vortexing for 2 min and subsequent centrifugation at 3,500 rpm for 10 min, the supernatant was collected for vacuum concentration until dry. The residue was reconstituted with 100 µL of 70% acetonitrile (in 5 mM ammonium acetate solution, pH 4.5), and 20 µL of the solution was injected for the determinations of SV and SVA via LC-MS/MS. As reported in our previous study, the linear range was 0.2–100 ng/ml for SV and 0.5–100 ng/ml for SVA with correlation coefficients (*r* ^2^) of 0.998 and 0.996, respectively.

Concentrations of GS in rat plasma samples were analyzed using an Agilent 1290 Ultrahigh Performance Liquid Chromatograph coupled to an Agilent 6430 Triple Quad (LC-MS/MS) with electrospray ionization (Agilent Technologies Inc., Santa Clara, CA, USA). Chromatographic separation was carried out in a Sunfire C_18_ (5 μm, 3 mm × 150 mm) column (Waters, MA, United States) by a mobile phase consisting of 0.1% formic acid (in distilled water) (A) and acetonitrile (B) with gradient elution (0.0 min, 20% B; 6.0 min, 90% B) at a flow rate of 0.5 ml/min. Selected ion monitoring (SIM) mode was applied to detect geniposide (*m/z* 411) and the internal standard (IS, paeoniflorin, *m/z* 503) with the MS condition: ion spray voltage at +4000 V; nitrogen as nebulizer at 30 psi; the gas flow rate at 8 L/min; and the Delta EMV at +400 V. For the preparation of the collected plasma samples of rats from HG1, HSG2, NG1, and NSG2 groups after the last dosing of GJ, 100 µL of each plasma sample after addition of 10 µL IS working solution was loaded onto a pre-conditioned (activated with 1 ml methanol and equilibrated with 1 ml water) Oasis^®^MCX cartridge followed by washing with 1 ml of water and elution with 1 ml methanol. The collected elution was vacuum concentrated to dry, and the residue was reconstituted with 50 µL of 20% acetonitrile in 0.1% formic acid with 20 µL of which injected for LC-MS/MS analysis of GS. The linearity range of the method for GS in rat plasma was 20–1,000 ng/ml with *r* ^2^ of 0.994.

### Effect of *Gardenia jasminoides* J. Ellis on the Hepatic mRNA and Protein Levels of Cyp2c11, Oatp1b2, and P-gp in Healthy and Nonalcoholic Steatohepatitis Rats Which Received Simvastatin

Total mRNA from livers of rats from groups HC, HS, HSG1, NC, NS, and NSG1 were extracted and purified using RNeasy kit (Qiagen, Venlo, Netherlands). After measuring the concentrations, reverse-transcription of mRNA to cDNA was conducted using a RevertAid First Strand cDNA synthesis kit (Thermo, Waltham, MA, USA). The mRNA levels of Mdr1a, Mdr1b, Oatp1b2, and Cyp2c11 were quantified using quantitative real-time PCR (LightCycler480II, Roche, Switzerland). Briefly, the cDNA samples (triplicates, 20 ng cDNA in each sample) were mixed with specific primers for the target gene and SYBR green detection system (Thermo, Waltham, MA, USA). The primer sequence of the genes was shown in Table 1. For each sample, 40 amplification cycles were conducted to obtain the cycle threshold (Ct), and the relative level of each target gene was calculated and presented as the ratio of GAPDH in the same sample using the formula: relative level = 2^−ΔCt^, in which ΔCt = Ct (target)–Ct (GAPDH).

**TABLE 1:** Primer sequences for quantitative real-time PCR.

Gene	5'→3′ forward	5'→3′ reverse
Mdr1a	CGT​TGC​CTA​CAT​CCA​GGT​TT	TGG​AGA​CGT​CAT​CTG​TGA​GC
Mdr1b	TGA​ATC​CCA​AAG​TGA​CAC​TGG	ATA​CTT​CTG​CGA​ATT​GAT​CTC​CTT​ATT
Oatp1b2	GGT​TGG​AAT​CGC​CAA​GTT​TGT	GGC​CAG​CAA​ATG​GCT​TGT​T
Cyp2c11	TGC​CCC​TTT​TTA​CGA​GGC​T	GGA​ACA​GAT​GAC​TCT​GAA​TTC​T
iNOS	GAC​CAG​AAA​CTG​TCT​CAC​CTG	CGA​ACA​TCG​AAC​GTC​TCA​CA
COX-1	GCT​CCA​GTT​TCC​CCC​TGC​T	TTC​TGG​CAT​GGA​TAG​TAA​CAA​CA
COX-2	ACCAACGCTGCCACAACT	GGT​TGG​AAC​AGC​AAG​GAT​TT
GAPDH	GGT​GGA​CCT​CAT​GGC​CTA​CAT	TGG​GTG​GTC​CAG​GGT​TTC​T

To extract total protein, the liver tissue of each rat from groups HC, HS, HSG1, NC, NS, and NSG1 was homogenized in RIPA cell lysis buffer which contains protease and phosphatase inhibitors followed by centrifugation at 15,000 rpm for 20 min at 4°C. The aliquots of each protein sample (20 μg) were separated using 8% SDS-polyacrylamide gel electrophoresis. After the proteins have been electrotransfered onto a polyvinylidene difluoride membrane (Millipore, Bedford, MA, United States), the membrane was blocked using 5% BSA in TBST and probed overnight at 4°C with the specific rabbit primary antibodies of P-gp (1:1,500), Oatp1b2 (1:1,500), Cyp2c11 (1:1,500), or GAPDH (1:2,500). After washing with TBST for three times, the membrane was incubated with goat anti-rabbit IgG second antibodies (1:5,000) at room temperature for 2 h followed by another three times of washing with TBST, and then the blots were detected using ChemiDoc MP Imaging System (Bio-Rad, Hercules, CA, United States) after exposure using ECL substrate (Bio-Rad, Hercules, CA, United States). The relative levels of targeting proteins in each sample were analyzed via normalizing the mean gray value of protein bands delimited in regions of interest by that of the GAPDH in each sample using ImageJ software (Bio-Rad, Hercules, CA, United States).

### Effect of Simvastatin on the P-gp–Mediated Efflux of Geniposide in MDCKII-MDR1 Cell Monolayer Model

MDCKII-MDR1 cells were cultured using Dulbecco’s modified Eagle medium (DMEM) supplemented with 10% fetal bovine serum (FBS), penicillin (100 U/mL), and streptomycin (100 μg/ml) in cell incubator (37°C, 5% CO_2_ with 90% relative humidity). To develop cell monolayer, MDCKII-MDR1 cells were seeded onto 12-well plate Transwell® inserts at a density of 1×10^5^ cells/well with 0.5 and 1.0 ml medium added to the apical (AP) and basolateral (BL) chamber, respectively. The cells were differentiated for 7 days with medium in AP and BL chambers refreshed every day. During the differentiation, transepithelial electrical resistance (TEER) was measured every day to monitor the integrity of the monolayer using Epithelial Voltammeter (EVOM-G, World Precision Instruments, Inc., Sarasota, FL, USA). MDCKII-MDR1 cell monolayer with TEER above 150 Ω cm^2^ was utilized in the transport studies (Brayden and Griffin, 2008). The experimental concentrations of SV and GS were chosen as 10 and 40 μM, respectively, due to that the ratio of SV (8.66 mg/kg) to the GS in the GJ (34.13 mg/kg) administered to the rats was close to 4. In addition, 20 µM SV has been found to be cytotoxic (viability of cells was <80%), while 10 µM SV was relatively safe (viability of cells was about 90.51%). Besides, verapamil (100 µM) was chosen as the positive compound for the inhibition of the P-gp–mediated transport. The cytotoxicities of GS (40 µM), GS (40 µM) with verapamil (100 µM), and GS (40 µM) with SV (10 µM) were tested in MDCKII-MDR1 cells with no significant cytotoxicity observed (Supplementary Material). Both AP-to-BL and BL-to-AP transports of GS (40 μM) were assessed in the absence and presence of verapamil (100 μM) or SV (10 μM). Briefly, the cell monolayers were preincubated with blank HBSS for 30 min, then the HBSS containing both GS and DMSO/verapamil/SV was added to the donor chamber (0.5 ml in AP and 1.0 ml in BL), and blank HBSS was added into the receiver chamber (1.0 ml in BL and 0.5 ml in AP), followed by collection of 100 μL sample from the receiver chamber every 0.5 h up to 2 h with refill of the same volume of blank HBSS. The collected samples were processed and analyzed using the same solid-phase extraction and LC-MS/MS method as described in LC-MS/MS quantification of SV, SVA, and/or GS in plasma of healthy and NASH rats to determine the concentration of GS.

### Effects of Simvastatin and/or *Gardenia jasminoides* J. Ellis on the Serum Biomarker Levels and Hepatic Histological Lesions in Healthy and Nonalcoholic Steatohepatitis Rats

ALT, AST, LDL/vLDL, and TC levels in the fasting serum collected of each rat on day 57 were measured using commercial kits following the manufacturer’s instruction, and three parallel repeats of each sample were performed with the mean value adopted. The embedded liver samples collected from each rat were sliced into 5 μm sections followed by deparaffinization, rehydration, and staining using hematoxylin and eosin (H&E). The liver sections of rats from groups HSG2, HG2, NSG2, and NG2 were also stained with Masson’s trichrome. The representative stained sections were observed by Eclipse Ti microscope with NIS-Elements Color Cam Ver. 4.00 (Nikon, Japan), and the hepatic histological lesions of each rat were semi-quantified based on the NAFLD Activity Score (NAS) (Kleiner et al., 2005).

### Detection of the GP-Protein Adduct in the Livers of the Rats Which Received *Gardenia jasminoides* J. Ellis Alone or in Combination With Simvastatin

Because GP was interacting with the L-lysine residue in protein to form the GP-protein adducts, such adducts needed to be processed to generate GP-lysine adduct for the detection. Chromatographic separation of the GP-lysine adducts was achieved using an Agilent 1290 Ultrahigh Performance Liquid Chromatograph with an ACQUITY UPLC BEC C_18_ (1.7 µM, 2.1 mm × 50 mm) column (Waters, MA, United States). The mobile phase consisted of 0.1 formic acid (A) and acetonitrile (B) with gradient elution (0 min, 10% B; 5 min, 20% B; 10 min, 20% B) at flow rate of 0.1 ml/min. The mass spectrometry was obtained using an Agilent 6430 Triple Quad with electrospray ionization in positive ionization model. Selected ion monitoring (SIM) model was used for the detection of the GP-lysine adducts ([M-CH_3_OH]^+^ *m/z* 305) with the MS conditions as ion spray voltage at +4000 V; nitrogen as nebulizer at 30 psi; the gas flow rate at 8 L/min, and the Delta EMV at +400 V.

For the identification of the GP-lysine adducts, 10 mM L-lysine was incubated with 1 mM GP (in PBS, pH 7.4) at 37°C for 3 h as the standard sample. In addition, 1 mM GP was incubated with 400 μL blank rat liver homogenate (healthy rats received no treatment, 1:2, w/v) at 37°C for 3 h to serve as the positive control. Meanwhile, the collected liver tissues of each rat from groups HC, HG1, HSG1, NC, NG1, and NSG1 were homogenized in PBS (1:2, w/v). The positive control sample and the liver homogenate samples were processed and digested using the method described in the previous study with a little modification (Li Z. et al., 2019). Briefly, 1.2 ml of iced methanol was added to 400 μL of each sample followed by vortexing and centrifugation at 4,000 g for 15 min. The pellets were collected and suspended in 1 ml denaturation buffer (5 mM DL-dithiothreitol in 8 M urea) followed by incubation at 37°C for 1 h. After centrifugation at 4,000 g for 15 min, the pellets were collected and suspended with 1 ml alkylation buffer (50 mM iodoacetamide in 8 M urea) with further incubation at 37°C for 1 h avoiding light. Thereafter, the mixtures were centrifuged at 4,000 g for 15 min with the pellet reconstituted in 1 ml 50 mM NH_4_HCO_3_ solution with protein concentration quantified and adjusted to 5 mg/ml. 0.5 ml of the sample was loaded into an Amicon Ultra-0.5 ml 3 K MWCO filter (EMD Millipore, Darmstadt, Germany) followed by centrifugation at 15,000 g for 15 min. The pellet was further washed with 0.5 ml of 50 mM NH_4_HCO_3_ solution for three times and then suspended in 250 μL of 50 mM NH_4_HCO_3_ solution. After incubation with 250 μL of protease (5 mg/ml) at 37°C for 8 h, the suspension was centrifuged at 15,000 g for 15 min, and the supernatant was collected for vacuum concentration to dryness followed by reconstitution in 50 μL 10% ACN (in 0.1% formic acid in water) for analysis using the LC-MS/MS method described above.

### Effects of Simvastatin and/or *Gardenia jasminoides* J. Ellis on the Hepatic mRNA and Protein Levels of iNOS, COX-1, and COX-2 in Healthy and Nonalcoholic Steatohepatitis Rats

Hepatic total mRNA and protein samples of each rat from groups HC, HS, HG1, HSG1, NC, NS, NG1, and NSG1 were extracted and processed as described in the effect of GJ on the hepatic mRNA and protein levels of Cyp2c11, Oatp1b2, and P-gp in healthy and NASH rats which received SV. mRNA levels of iNOS, COX-1, and COX-2 were quantified using quantitative real-time PCR (LightCycler480II, Roche, Switzerland). The primer sequences of the target genes are shown in Table 1. For each sample, 40 amplification cycles were conducted to obtain the cycle threshold (Ct), and the relative level of each target gene was calculated and presented as the ratio of GAPDH from sample using the formula: relative level = 2^−ΔCt^, in which ΔCt = Ct (target)–Ct (GAPDH). Aliquots of each protein sample (20 μg) were separated using 8% SDS-polyacrylamide gel electrophoresis. After the proteins have been electrotransfered onto a polyvinylidene difluoride membrane (Millipore, Bedford, MA, United States), the membrane was blocked using 5% BSA in TBST and probed overnight at 4°C with the specific rabbit anti-primary antibodies of iNOS (1:1,000), COX-1 (1:1,000), COX-2 (1:1,000), or GAPDH (1:2,500). After washing with TBST for three times, the membrane was incubated with goat anti-rabbit IgG second antibodies (1:5,000) at room temperature for 2 h followed by another three times of washing with TBST, and then the blots were detected using ChemiDoc MP Imaging System (Bio-Rad, Hercules, CA, United States) after exposure with ECL substrate (Bio-Rad, Hercules, CA, United States). All the primary and secondary antibodies were diluted using 5% BSA in TBST.

### Data analyses

The plasma concentration vs. time profiles of SV, SVA, and GS were analyzed using non-compartmental model by WinNonlin software (Phoenix 64, Certara, NJ, United States). The peak plasma concentration (C_max_) and the time to reach C_max_ (T_max_) were obtained directly from the profiles. Area under the plasma concentration vs. time curve from zero to infinity and from zero to the last time point (AUC_0-∞_ and AUC_0-last_), apparent plasma clearance (CL/F), and terminal elimination half-life (T_1/2_) was assessed if applicable. The fold of change for the relative mRNA and protein levels in rats from each group were obtained after standardization to the mean value of that in healthy or NASH rats.

The permeability coefficient (*P*_app_) of GS in the MDCKII-MDR1 cell monolayer model was calculated by the following equation:

$$P_{\mathrm{app}}=\frac{dQ\cdot V}{dt}\cdot \frac{1}{A\cdot C_{0}}.$$

*dQ/dt* is the accumulation rate of the GS in the receiver chamber over time (µM/s); V is the volume of the solution in receiver chamber (ml); A is the membrane surface area (cm^2^); and C_0_ is the initial concentration of GS in the donor chamber (µM).

Statistically significant differences in the pharmacokinetic parameters, histological semi-quantification results, serum biomarker levels, relative mRNA levels, and relative protein levels between healthy and NASH rats which received different drug/herb/vehicle treatments were analyzed using one-way ANOVA followed by Tukey’s post hoc test performed with SPSS Statistics 24.0.0.0 (IBM Corporation, Armonk, New York, United States) with *p < 0.05, **p < 0.01, and ***p < 0.001 considered to be significantly different. All the results were expressed as the mean ± standard deviation.

## Results

### More Significant Pharmacokinetic Interactions Between Simvastatin and *Gardenia jasminoides* J. Ellis in Healthy Rats Than That in Nonalcoholic Steatohepatitis Rats

**FIGURE 3:**
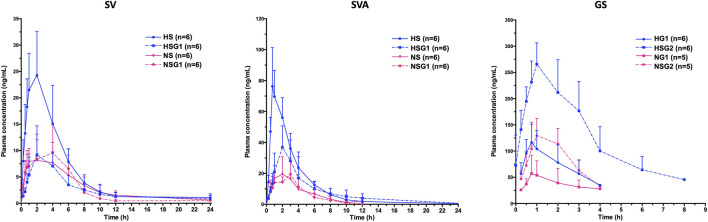
Pharmacokinetic profiles of SV and SVA in healthy and NASH rats which received SV in the absence (HS and NS) and presence (HSG1 and NSG1) of GJ and those of GS in healthy and NASH rats which received GJ in the absence (HG1 and NG1) and presence (HSG2 and NSG2) of SV. H and N denoted healthy and NASH, respectively. C, S, and G indicated that the rats received vehicle, SV, and GJ, respectively.

The pharmacokinetic profiles of SV, SVA, and GS in healthy and NASH rats after oral administration of SV, GJ, and their combination are illustrated in Figure 3. In healthy rats, the pharmacokinetic parameters shown in Table 2 indicated that C_max_ and AUC_0-∞_ of both SV and SVA in the rats from group HSG1 were significantly lower than those from group HS, and the CL/F of SV in the rats from group HSG1 was significantly higher than that from group HS. The results demonstrated that coadministration with GJ significantly reduced the systemic exposures of both SV and SVA in healthy rats. While in NASH rats, all the pharmacokinetic parameters of SV and SVA in the rats from NS were not significantly differed from those from group NSG1 (Table 2), which demonstrated that coadministration with GJ did not influence the pharmacokinetics of SV and SVA in NASH rats, which differed from what has been observed for healthy rats. Moreover, the absolute systemic exposure of SV was in the order as healthy rats received SV (HS) > healthy rats received SV with GJ (HSG1) ≈ NASH rats received SV (NS) ≈ NASH rats received SV with GJ (NSG1). The order of SVA was healthy rats received SV (HS) > healthy rats received SV with GJ (HSG1) ≳ NASH rats received SV (NS) ≈ NASH rats received SV with GJ (NSG1).

**TABLE 2:** Pharmacokinetic (PK) parameters of SV, SVA, and GS in healthy and NASH rats which received SV (8.66 mg/kg, p.o., once daily), GJ (325 mg/kg, p.o., twice daily), and their combination.

PK parameters	SV				SVA				GS			
	Healthy rats		NASH rats		Healthy rats		NASH rats		Healthy rats		NASH rats	
	HS (*n* = 6,^#^)	HSG1 (*n* = 6, ^$^)	NS (*n* = 6, ^^^)	NSG1 (*n* = 6)	HS (*n* = 6,^#^)	HSG1 (*n* = 6, ^$^)	NS (*n* = 6, ^^^)	NSG1 (*n* = 6)	HG1 (*n* = 5,^#^)	HSG2 (*n* = 5, ^$^)	NG1 (*n* = 5, ^^^)	NSG2 (*n* = 5)
C_max_ (ng/ml	27.92 ± 6.27	11.56 ± 3.99^##^	9.74 ± 3.17^###^	12.63 ± 3.71^##^	82.49 ± 18.70	44.26 ± 8.30^##^	24.00 ± 12.60^#^	23.70 ± 7.42^$$##^	136.29 ± 26.98	267.08 ± 38.43^###^	61.93 ± 29.30^##^	141.66 ± 40.70^^^$$$^
T_max_ (h)	1.79 ± 0.81	2.00 ± 0.89	1.62 ± 1.24	2.04 ± 1.16	1.00 ± 0.50	2.33 ± 1.03	1.45 ± 0.87	2.50 ± 1.22	0.88 ± 0.21	0.96 ± 0.10	0.85 ± 0.14	1.15 ± 0.49
AUC_0-∞_ (h*ng/mL)	95.04 ± 21.27	45.24 ± 10.15^##^	48.53 ± 21.06^##^	43.21 ± 15.39^##^	265.47 ± 43.07	180.09 ± 71.81^#^	92.41 ± 42.51^#^	82.99 ± 31.23^$##^	—	—	—	—
AUC_0-last_ (h*ng/mL)	93.59 ± 19.38	42.42 ± 9.27^##^	46.28 ± 19.80^##^	42.55 ± 15.38^##^	263.81 ± 42.09	175.71 ± 71.91^#^	89.57 ± 41.74^#^	81.65 ± 30.09^$##^	257.21 ± 107.13	904.27 ± 202.97^###^	114.91 ± 81.02^#^	320.86 ± 72.56^^^$$$^
CL/F (L/h/kg	95.00 ± 22.32	199.74 ± 45.80^##^	231.70 ± 161.99^###^	223.45 ± 78.85^###^	—	—	—	—	—	337.1 ± 123.30	—	—
T_1/2_ (h)	2.06 ± 0.96	3.85 ± 1.69	2.15 ± 1.00	1.44 ± 0.51	2.05 ± 1.50	3.14 ± 1.18	1.86 ± 0.91	1.64 ± 0.28	—	1.85 ± 1.18	—	—

For SV and SVA, ^#^ *p*< 0.05, ^##^ *p*< 0.01, and ^###^ *p*< 0.001 compared to rats from HS; ^$^ *p*< 0.05, ^$$^ *p*< 0.01, and ^$$$^ *p*< 0.001 compared to rats from HSG1; ^^^ *p*< 0.05, ^^^^ *p*< 0.01, and ^^^^^ *p*< 0.001 compared to rats from NS.

For GS, ^#^ *p*< 0.05, ^##^ *p*< 0.01, and ^###^ *p*< 0.001 compared to rats from HG1; ^$^ *p*< 0.05, ^$$^ *p*< 0.01, and ^$$$^ *p*< 0.001 compared to rats from HSG2; ^^^ *p*< 0.05, ^^^^ *p*< 0.01, and ^^^^^ *p*< 0.001 compared to rats from NG1.

H and N denoted healthy and NASH, respectively. C, S, and G indicated that the rats received vehicle, SV, and GJ, respectively.

In healthy rats, the plasma C_max_ and AUC_0-∞_ of GS in the rats from HSG2 were 1.95 and 3.52 times of those from group HG1, respectively (*p* < 0.05). It was indicated that coadministration with SV significantly increased the systemic exposure of GS in healthy rats. In NASH rats, the plasma C_max_ and AUC_0-∞_ of GS in the rats from NSG2 were 2.28- and 2.79-fold of those in the rats from NG1, respectively. The T_max_ of GS was similar between the two groups, which suggested that coadministration of SV significantly increased the systemic exposure of GS in NASH rats, which was consistent with the result observed in healthy rats. However, due to the incomplete elimination phase of GS in the rats from groups HG1, NG1, and NSG2, the CL/F and T_1/2_ of GS were not available for further comparison. The systemic exposures of GS were in the order of healthy rats received SV with GJ (HSG2) > healthy rats received GJ (HG1) ≈ NASH rats received SV with GJ (NSG2) > NASH rats received GJ (NG1).

### Up-Regulated P-gp mRNA and Protein Expressions by Coadministration of *Gardenia jasminoides* J. Ellis in Healthy Rats but Not Nonalcoholic Steatohepatitis Rats

**FIGURE 4:**
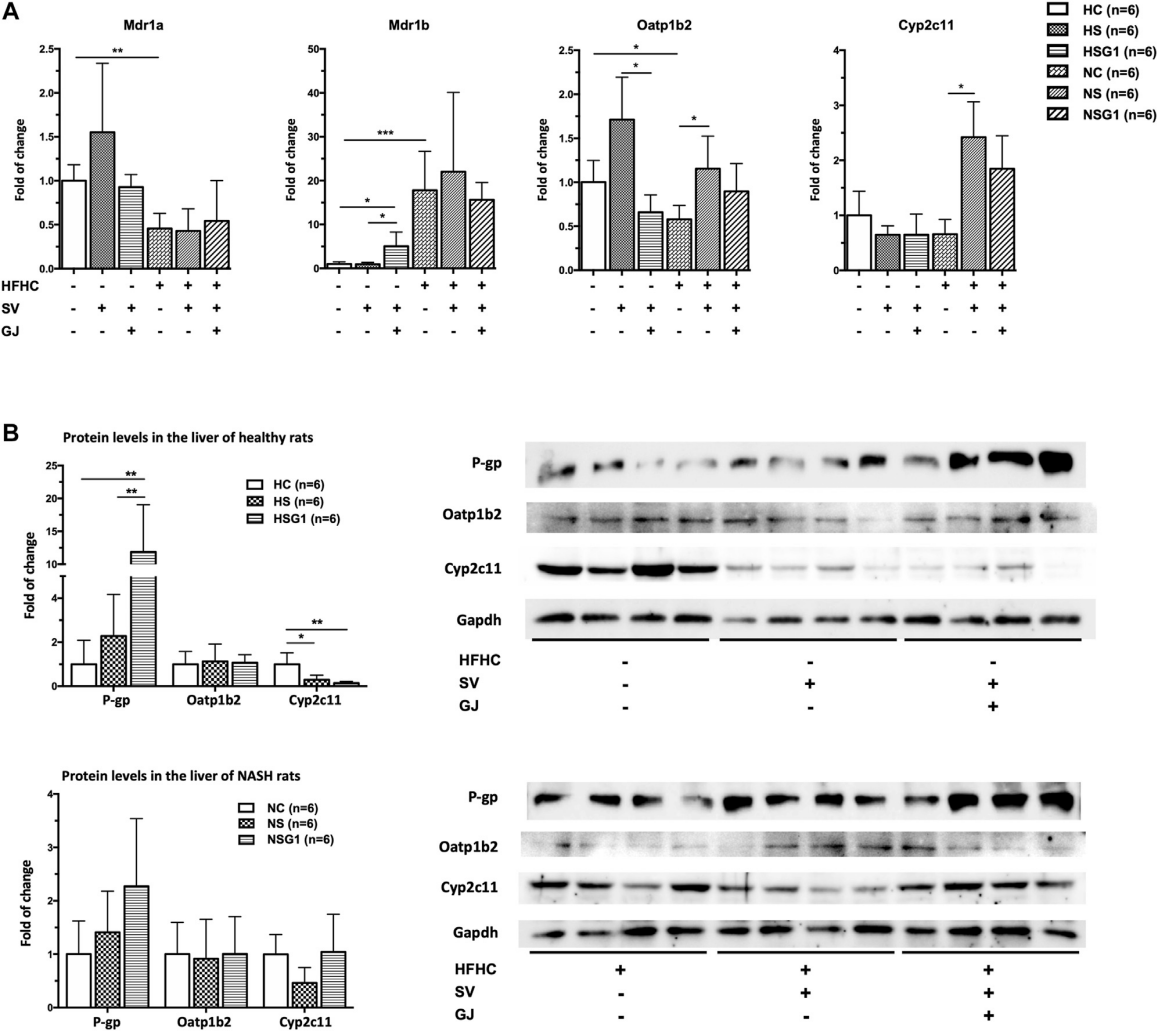
Hepatic mRNA **(A)** and protein **(B)** levels of Mdr1a/1 b (P-gp), Oatp1b2, and Cyp2c11 in healthy and NASH rats which received vehicle (HC and NC), SV (HS and NS), and SV and GJ (HSG1 and NSG1) with representative bands. H and N denoted healthy and NASH, respectively. C, S, and G indicated that the rats received vehicle, SV, and GJ, respectively. *p < 0.05, **p < 0.01, and ***p < 0.001.

To investigate the underlying mechanism of the effects of GJ on the pharmacokinetics of SV and SVA in both healthy and NASH rats, the mRNA and protein levels of hepatic P-gp, Oatp1b2, and Cyp2c11 in rats from groups HC, HS, HSG1, NC, NS, and NSG1 were detected, as shown in Figure 4A. In healthy rats, two weeks treatment of SV (HS) showed no significant influence on the hepatic mRNA levels of Mdr1a, Mdr1b, Oatp1b2, and Cyp2c11. In addition, two weeks co-treatment of GJ with SV (HSG1) significantly induced the mRNA level of Mdr1b for about 5.08-fold, reduced the mRNA level of Oatp1b2 for around 61.54%, and did not change the mRNA levels of Mdr1a and Cyp2c11 compared to the treatment of SV in healthy rats (HS). While in NASH rats, two weeks treatment of SV (NS) significantly increased the mRNA levels of Oatp1b2 and Cyp2c11 for 1.99- and 3.68-fold compared to the vehicle (NC), respectively, while did not influence the mRNA levels of Mdr1a and Mdr1b. Meanwhile, coadministration of SV and GJ (NSG1) showed no further significant influence on the mRNA levels of Mdr1a, Mdr1b, Oatp1b2, and Cyp2c11 compared to the administration with SV to NASH rats (NS).

In healthy rats, two weeks administration of SV (HS) did not influence the hepatic protein levels of P-gp and Oatp1b2 but significantly reduced the hepatic protein level of Cyp2c11 for 71.50% (*p* < 0.05) compared to the treatment of vehicle (HC), as shown in Figure 4B. Moreover, SV and GJ coadministration (HSG1) significantly elevated the hepatic P-gp protein level in healthy rats compared to the administration of SV (HS), while the protein levels of hepatic Oatp1b2 and Cyp2c11 were similar between rats from groups HSG1 and HS. On the other hand, the administrations of SV in the absence and presence of GJ (NS and NSG1) showed no significant effects on the hepatic protein levels of P-gp, Oatp1b2, and Cyp2c11 in NASH rats.

### Inhibition of Simvastatin on the P-gp–Mediated Transport of Geniposide in MDCKII-MDR1 Monolayer Model

After 7 days of differentiation, TEER of the MDCKII-MDR1 monolayer reached up to 230–250 Ω cm^2^. In such cell monolayer, the P_app(AP-BL)_ and P_app(BL-AP)_ of GS (40 µM) were 4.73 ± 0.41 × 10^−6^ cm/s and 8.89 ± 0.36 × 10^–6^ cm/s, respectively, and the efflux ratio (P_app(BL-AP)_/P_app(AP-BL)_) was 1.88 (Table 3), indicating the existence of active efflux transport of GS. In the presence of verapamil (100 µM), the P_app(AP-BL)_ of GS remained similar, whereas the P_app(BL-AP)_ was significantly decreased, leading to a lower efflux ratio (1.27). Similarly, compared to the groups which received GS alone, the presence of SV significantly reduced the P_app(BL-AP)_ of GS while showed no significant effects on the P_app(AP-BL)_ of GS, and the efflux ratio of GS was lower (1.32).

**TABLE 3:** Effects of verapamil (100 µM) and SV (10 µM) on the transport of GS (40 µM) in the MDCKII-MDR1 monolayer cell model (*n* = 3).

Groups	P_app_ (10^–6^ cm/s)		P_app(BL-AP)_/P_app(AP-BL)_
	AP-BL	BL-AP	
GS (40 µM)	4.73 ± 0.41	8.89 ± 0.36	1.88
GS (40 µM) + verapamil (100 µM)	5.82 ± 0.89	7.37 ± 0.57^a^	1.27
GS (40 µM) + SV (10 µM)	5.03 ± 0.27	6.64 ± 0.41^b^	1.32

^a^ *p*< 0.05.

^b^ *p*< 0.01 compared to the GS (40 µM) treated group.

### Coadministration of Simvastatin and *Gardenia jasminoides* J. Ellis Induced Hepatotoxicity in Healthy Rats and Hepatoprotective Effects in Nonalcoholic Steatohepatitis Rats

**FIGURE 5:**
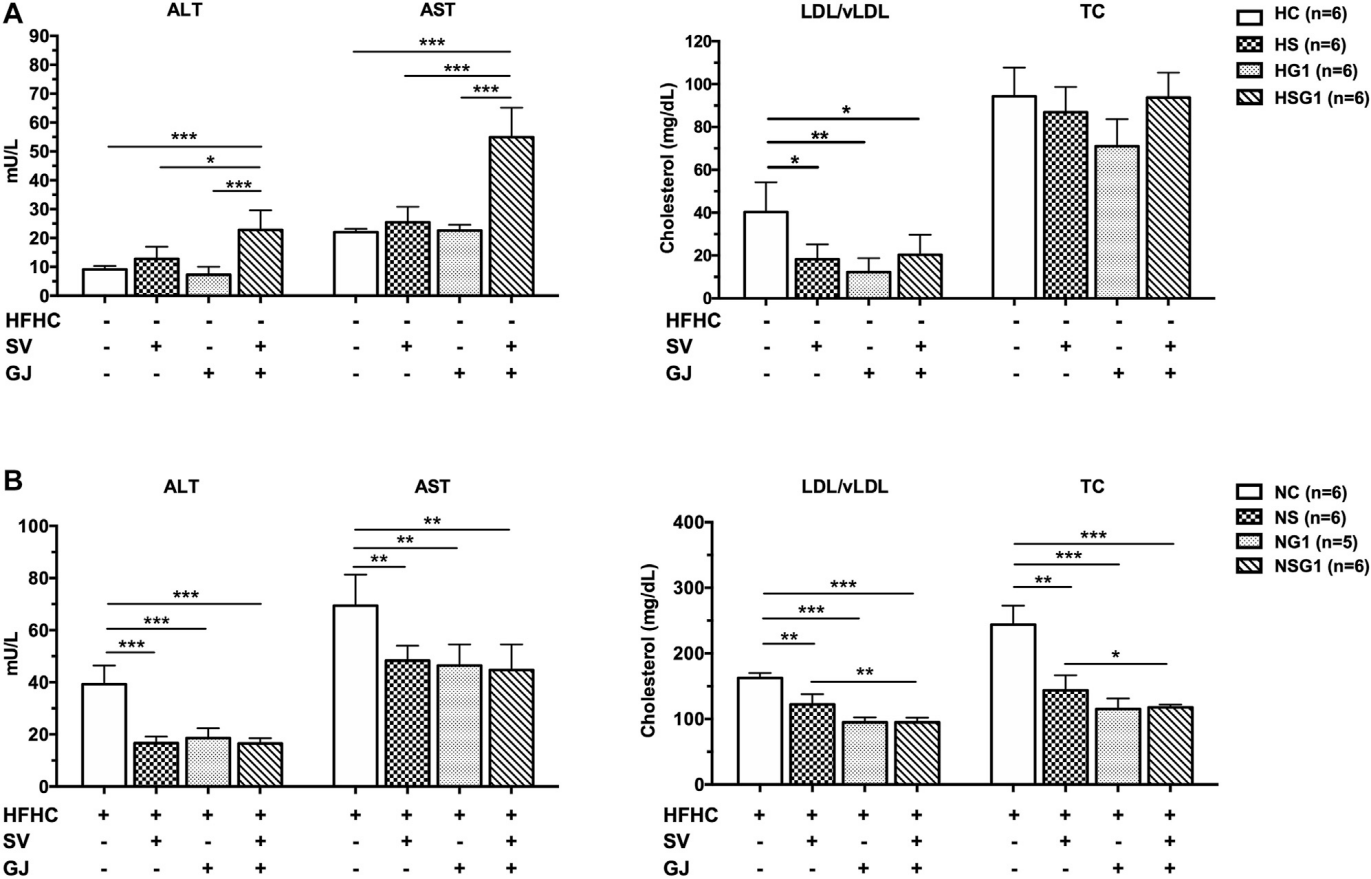
ALT, AST, LDL/vLDL, and TC levels in the fasting serum of healthy **(A)** and NASH **(B)** rats after two-week treatments of vehicle (HC and NC), SV (HS and NS), GJ (HG1 and NG1), and their combination treatment (HSG1 and NSG1). H and N denoted healthy and NASH, respectively. C, S, and G indicated that the rats received vehicle, SV, and GJ, respectively.

As shown in Figure 5A, serum ALT and AST levels in the rats from groups HS and HG1 were not significantly different from those from group HC. Nevertheless, significant higher ALT and AST levels in rats from group HSG1 (22.79 ± 6.78 mU/L and 54.96 ± 10.18 mU/L) compared to those in rats from group HC (9.14 ± 1.10 mU/L and 22.02 ± 1.11 mU/L) were observed. The results indicated that oral administration of SV or GJ for two weeks could not induce liver injury in healthy rats, but their combination was hepatotoxic. Besides, compared to rats from group HC, two-week oral administration of SV (HS), GJ (HG1), and their combination (HSG1) all significantly reduced the LDL/vLDL level to a similar extent but showed no significant impact on the TC level in healthy rats. In NASH rats, two-week treatment of SV (NS) and GJ (NG1) significantly reduced the serum ALT (42.46 and 47.34%), AST (69.65 and 66.98%), LDL/vLDL (75.21 and 58.36%), and TC (58.95 and 48.27%) levels compared to the treatment of vehicle (NC) (Figure 5B). Their coadministration in NASH rats (NSG1) also lowered the serum ALT (42.12%), AST (64.44%), LDL/vLDL (58.35%), and TC (47.26%) compared to the treatment of vehicle (NC).

**FIGURE 6:**
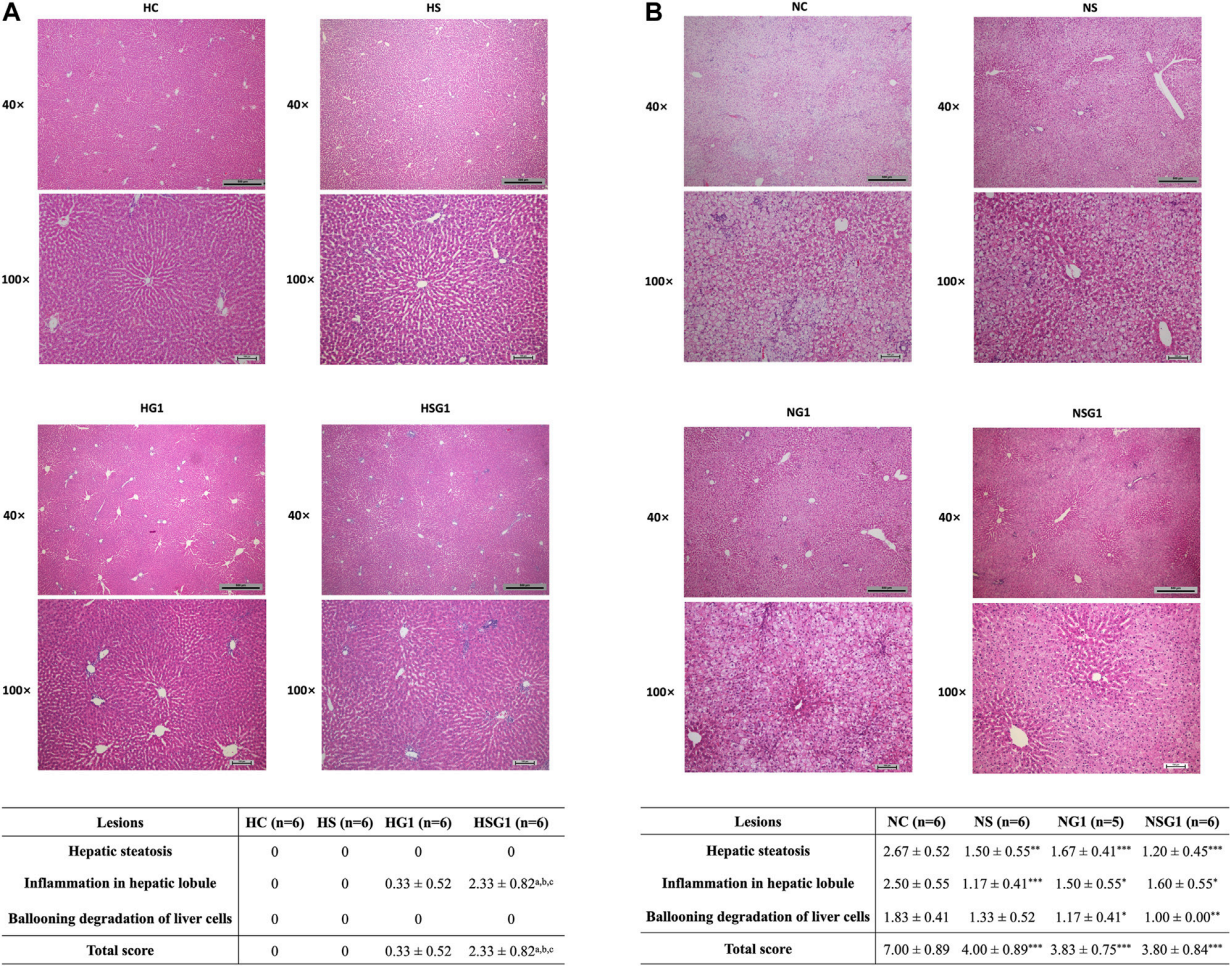
Histological evaluation by H&E staining of livers from healthy **(A)** and NASH **(B)** rats which received two-week vehicle (HC and NC), SV (HS and NS), GJ (HG1 and NG1), and their combination treatment (HSG1 and NSG1) and their NAS-based semi-quantification. H and N denoted healthy and NASH, respectively. C, S, and G indicated that the rats received vehicle, SV, and GJ, respectively. ^a^ *p* < 0.001, ^b^ *p*<0.001, and ^c^ *p*<0.001 compared to the rats from groups HC, HS, and HG1, respectively. ^***^ *p* < 0.001 compared to the rats from the NC group.

H&E staining results showed that two-week administration of SV did not induce any significant lesion in the livers of healthy rats (HS) (Figure 6A). Meanwhile, two-week treatment of GJ resulted in mild inflammation infiltration in the liver of healthy rats (HG1) (*p* > 0.05). Importantly, inflammation infiltration in the liver of healthy rats which received coadministration of SV and GJ (HSG1) was more severe than the healthy rats which received vehicle (HC), SV (HS), and GJ (HG1) (*p* < 0.05). On the other hand, both steatosis and inflammation infiltration in the liver of NASH rats were significantly alleviated by the treatment of SV (NS), GJ (NG1), and their combination (NSG1) (Figure 6B). Nevertheless, there were no synergistic effects of such protective effects of SV and GJ observed. Meanwhile, the treatment of GJ (NG1) and coadministration of SV with GJ (NSG1) significantly attenuated the ballooning degradation of hepatocytes, while the treatment of SV (NS) did not exert such protection.

### Significant Formation of Hepatic GP-Protein Adducts in Healthy Rats Coadministered with Simvastatin and *Gardenia jasminoides* J. Ellis

**FIGURE 7:**
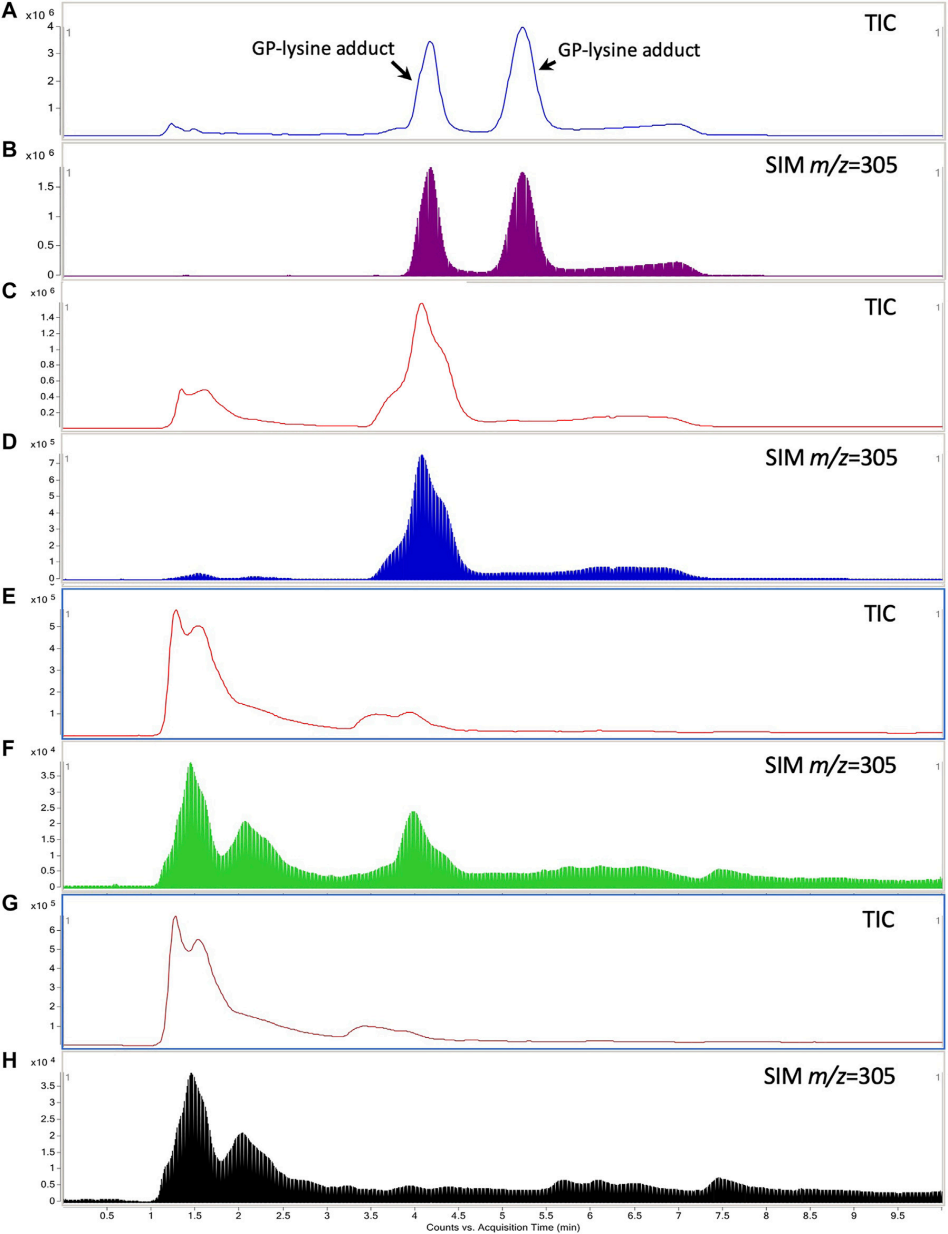
Typical total ion chromatogram (TIC) and extracted ion chromatography (*m/z* = 305) of the GP-lysine adduct in the incubation mixture of 10 mM L-lysine with 1 mM GP **(A, B)**, mixture of blank liver homogenate with 1 mM GP after incubation **(C, D)**, liver homogenate of healthy rat which received two-week coadministration of SV and GJ **(E, F)**, and liver homogenate of healthy rat which received two-week administration of GJ **(G, H)**.

Two GP-lysine adducts were detected in the mixture of GP (10 mM) and L-lysine (1 mM) incubated at 37°C for 3 h with retention times of 4.26 and 5.32 min, respectively (Figures 7A,B). Such observation was consistent with the results of the previous study (Li Y. et al., 2019). Only one of the two GP-lysine adducts was detected in the incubation mixture of blank liver homogenate and GP (Figures 7C,D), which was expected due to the fact that only one primary amine in the residue of L-lysine in protein was available for the interaction with GP (Li Y. et al., 2019). As shown in Figures 7E–H, the hepatotoxic GP-lysine adduct would only be detected in the liver homogenate of healthy rats which received two-week coadministration of SV and GJ (HSG1) and was barely detectable in the liver homogenate of healthy rats which received GJ (HG1) and that of NASH rats which received GJ in absence and presence of SV (NG1 and NSG1).

### Coadministration of Simvastatin and *Gardenia jasminoides* J. Ellis Lead to Inflammation-Promotion in Healthy Rats and Inflammation-Suppression in Nonalcoholic Steatohepatitis Rats

**FIGURE 8:**
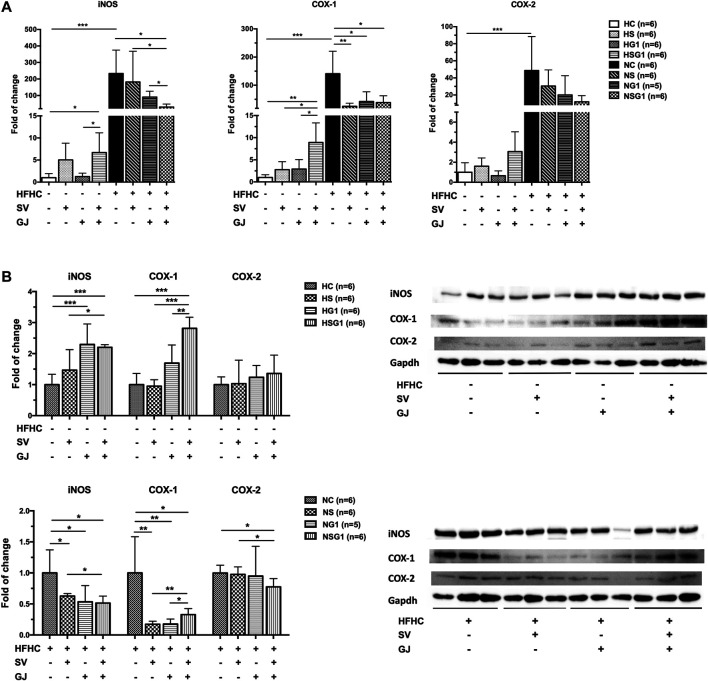
Hepatic mRNA **(A)** and protein **(B)** levels of iNOS, COX-1, and COX-2 in healthy and NASH rats which received vehicle (HC and NC), SV (HS and NS), GJ (HG1 and NG1), and their combination (HSG1 and NSG1) with representative bands. H and N denoted healthy and NASH, respectively. C, S, and G indicated that the rats received vehicle, SV, and GJ, respectively. *p < 0.05, **p < 0.01, and ***p < 0.001.

In healthy rats, treatment of SV (HS) and GJ (HG1) did not significantly affect the hepatic iNOS, COX-1, and COX-2 mRNA levels as indicated in Figure 8A. However, coadministration of SV and GJ (HSG1) up-regulated the mRNA expressions of iNOS and COX-1 (*p* < 0.05) and showed a trend of elevating the mRNA level of COX-2 (*p* > 0.05). Compared to the healthy control (HC), NASH rats (NC) showed significantly higher hepatic mRNA levels of iNOS, COX-1, and COX-2. Meanwhile, two-week treatment of SV (NS) and GJ (NG1) both significantly reduced the COX-1 mRNA level and did not change the iNOS and COX-2 mRNA levels. NASH rats received coadministration of SV and GJ (NSG1) which showed lower hepatic iNOS (*p* < 0.05), COX-1 (*p* < 0.05), and COX-2 (*p* = 0.0505) mRNA levels compared to those from group NC. Additionally, the hepatic iNOS mRNA level in rats from group NSG1 was significantly lower compared to those from groups NS and NG1.

As shown in Figure 8B, two-week treatment of SV (HS) showed no significant influence on the hepatic protein levels of iNOS, COX-1, and COX-2 in healthy rats, while the treatment of GJ (HG1) increased the protein level of iNOS for about 2.29-fold (*p* < 0.05). Importantly, the hepatic protein levels of iNOS and COX-1 were significantly elevated for 2.20- and 2.81-fold in healthy rats which received co-treatment of SV and GJ (HSG1), respectively. On the other hand, in the liver of NASH rats which received SV (NS), GJ (NG1), and their combination (NSG1), the iNOS and COX-1 levels were significantly lower (62.72, 47.58, and 51.49% for iNOS and 17.32, 20.08, and 32.75% for COX-1) than those in the NASH rats which received vehicle (NC). In addition, SV and GJ coadministration significantly reduced the protein level of COX-2 for 22.42% (*p* < 0.05), while neither administration of SV (NS) nor GJ (NG1) showed significant effects on the protein level of COX-2.

### Irreversible Liver Injury Induced by Two-Week Coadministration of Simvastatin and *Gardenia jasminoides* J. Ellis in Healthy and Nonalcoholic Steatohepatitis Rats

**FIGURE 9:**
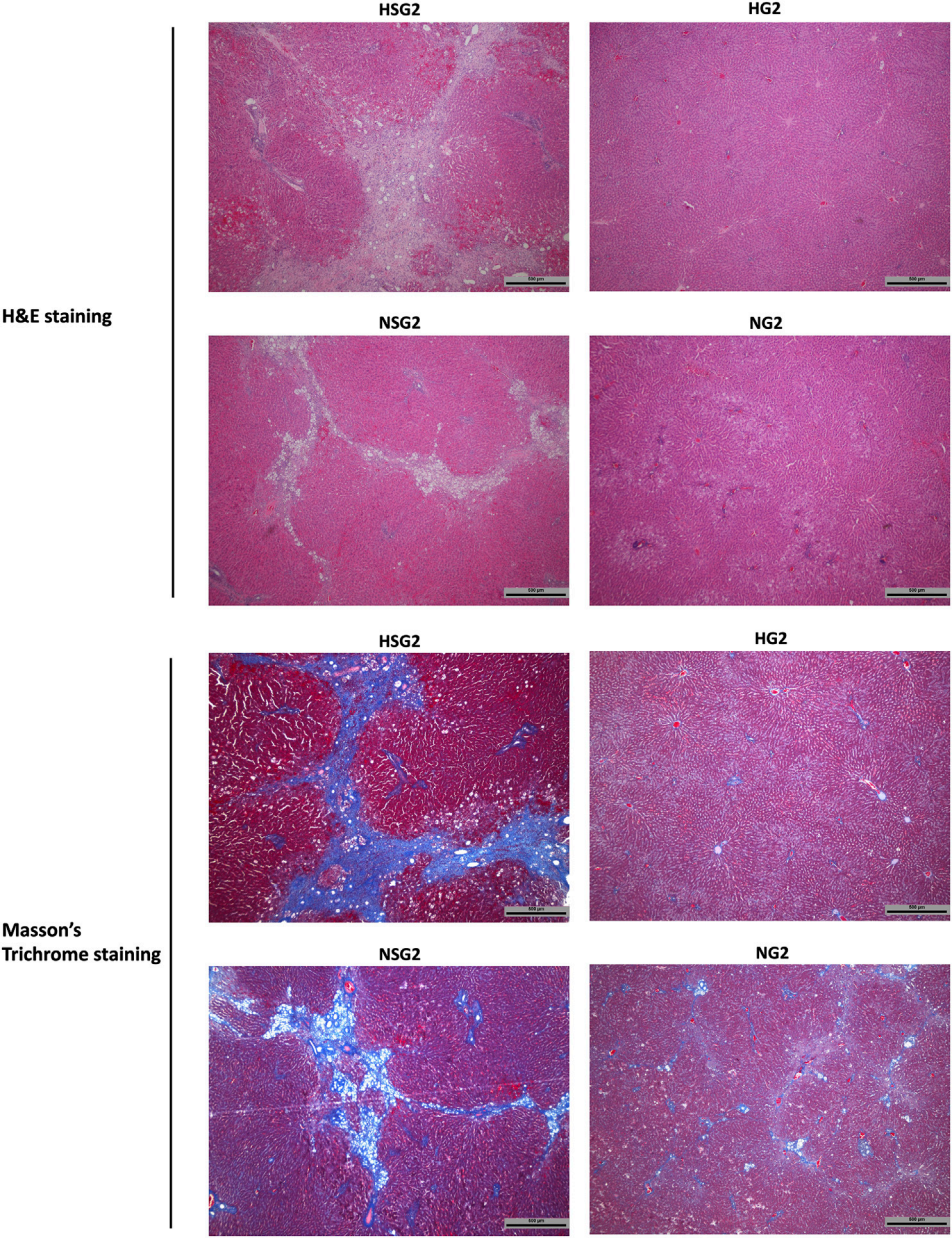
Hepatic H&E and Masson’s trichrome staining of healthy and NASH rats which received two-week coadministration of SV and GJ (HSG2 and NSG2) and GJ (HG2 and NG2) at 90 days after discontinuation of herb/drug treatments. H and N denoted healthy and NASH, respectively. C, S, and G indicated that the rats received vehicle, SV, and GJ, respectively.

At day 90 after discontinuation of herb/drug treatment, the livers of healthy rats which received two-week SV and GJ coadministration (HSG2) showed severe inflammation infiltration, ballooning, and fibrosis as indicated by the H&E and Masson’s trichrome stained liver slices (Figure 9), while only slight inflammation infiltration was observed in the liver of healthy rats ever treated with two-week GJ (HG2). Similar to the healthy rats, NASH rats which received two-week coadministration of SV and GJ (NSG2) showed more severe ballooning and fibrosis compared to those which received GJ (NG2). Moreover, the degree of fibrosis in rats from group NSG2 was less severe than that from group HSG2.

## Discussion

Our present study investigated the interactions between SV and GJ in healthy and NASH rats from both pharmacokinetic and pharmacodynamic aspects. For pharmacokinetic interactions, we monitored the concentration changes of SV, SVA, and GS (the major absorbable component in GJ) in healthy and NASH rats which received SV and/or GJ. Although sulfate and glucuronide of GP were reported as the metabolites after oral administration of GJ (Hou et al., 2008; Zhang et al., 2017), they were barely detectable in our collected plasma samples (no GP detected before and after hydrolyzing with sulfatase and β-glucuronidase). For pharmacodynamic interactions, we detected and compared the liver functions and lipid levels among the relevant treatment groups. Our findings indicated that the interactions between SV and GJ could result in very different pharmacodynamic outcomes in healthy and NASH rats, which were related to the different pharmacokinetic interactions.

In healthy rats, treatment of SV slightly elevated the serum ALT and AST levels and treatment of GJ resulted in not significant hepatic inflammation infiltration at their clinical relevant dose. By comparison, their coadministration significantly increased the serum ALT and AST levels and aggravated the hepatic inflammation infiltration, demonstrating that interaction between SV and GJ could result in hepatotoxicity in healthy rats. It was noticed that coadministration of SV and GJ in healthy rats reduced the systemic exposures of SV and SVA, while increasing the systemic exposure of GS. GS was deemed hepatotoxic at high dose because its metabolite GP could interact with the proteins in the liver to form GP-protein adduct, therefore, inducing liver injury (Ding et al., 2013; Li Y. et al., 2019). In this context, the interaction between SV and GJ induced hepatotoxicity was suggested to be attributed to the increased systemic exposure of GS, which was supported by the higher content of GP-protein adducts detected in the liver. In addition, treatments of SV, GJ, and their combination all decreased the serum LDL/vLDL level but not the TC level in healthy rats. It is well-known that SV could alleviate the serum LDL level, while the LDL/vLDL-lowering effect of GJ in healthy rats has never been reported before.

In the exploration on the mechanism of the pharmacokinetic interactions in healthy rats, our findings indicated that SV could inhibit the P-gp–mediated efflux of GS, demonstrating that coadministration with SV could increase the systemic exposure of GS via reducing the intestinal P-gp–mediated efflux during its absorption because GS is a substrate of P-gp (Yu et al., 2017). The inhibition of SV on the transport of various P-gp substrates in different cell models including Caco-2, CR1R12, and 3T3-G18 has been previously reported (Wang et al., 2001). In addition, increased systemic exposure of GS induced by coadministration of herbs/drugs has been previously reported in several studies despite unclear mechanism (Cheng et al., 2014). However, due to limited available ADME information of GJ and GS, the effect of SV on the process other than P-gp–mediated intestinal efflux of GS remained unknown in the current study. On the other hand, coadministration of GJ decreased the systemic exposures of SV and SVA in healthy rats via up-regulating the hepatic expression of P-gp, which could promote the biliary efflux of SVA. Due to that GP could increase the P-gp expression in HepG2 cells (Gao et al., 2014), such up-regulation on the hepatic P-gp expression of coadministration of GJ was suggested to be induced by GP that generated in the liver by hydrolysis of GS. In addition, both GS and GP possess choleretic activities, implying that the coadministration of GJ may accelerate the secretion of bile, therefore, facilitating the biliary excretion of both SV and SVA (Shoda et al., 2004; Wang et al., 2017). Besides, given that the protein level of hepatic Cyp2c11 has been significantly down-regulated by the treatment of SV, the influence of GJ on the protein level of Cyp2c11 was not significant, although GP has been reported to be able to reduce the expression of CYP2C19 (isoform of Cyp2c11 in human) in HepG2 cell as well (Gao et al., 2014). Moreover, the significant down-regulation on the Cyp2c11 expression that resulted from two-week SV treatment was firstly reported in the present study, which might lead to less metabolism of not only SV but also co-used drug/herb who is a substrate of Cyp2c11.

In NASH rats, both SV and GJ treatments exerted significant hepatoprotective and lipid-lowering activities. Such beneficial effects of them on NAFLD/NASH patients/animal models have been previously reported (Abel et al., 2009; Nelson et al., 2009; Ma et al., 2011). Besides, our results demonstrated that the coadministration of them showed synergistic effect on their lipid-lowering activity but not hepatoprotective effects. The attenuated hepatic inflammation in NASH rats which received SV and/or GJ shown in the liver histology demonstrated that the hepatoprotective activities of them and their combination might be attributed to anti-inflammation. Interestingly, SV and GJ coadministration enhanced the alleviation on the expression of iNOS and COX-2 of SV but reduced the reduction on the expression of iNOS of SV and GJ, which might explain the unchanged hepatoprotective effect of the coadministration of SV and GJ compared to the administration of either of them. Although the anti-inflammatory activities of both SV and GJ have been previously recognized in NASH and other diseases (Koo et al., 2006; Abel et al., 2009; Ma et al., 2011; Al-Rasheed et al., 2017), the anti-inflammation of their combination is firstly observed in the current study. Moreover, the beneficial activities in NASH rats resulted from SV and GJ coadministration were in contrast to the hepatotoxicity in healthy rats which might be induced by the high systemic exposure of GS and content of GP-protein adducts. In NASH rats which received SV and GJ co-treatment, the systemic exposure of GS was lower compared to that in healthy rats, and the hepatic GP-protein adducts were barely detectable.

In NASH rats, coadministration with SV increased the systemic exposure of GS, which was suggested to result from the suppression on the P-gp–mediated efflux of GS induced by SV similar to what happened in healthy rats. It is also worth to note that the absolute AUC_0-last_ of GS in NASH rats was lower than that in the healthy rats after administration of GJ alone and in combination with simvastatin, respectively. Such alteration was similar to the lower systemic exposure of SV and SVA after oral administration of SV in NASH rats. Given that GS was majorly absorbed via passive diffusion and also a substrate of P-gp (Yu et al., 2017), it was suggested that the intestinal and hepatic histological and pharmacological alterations in NASH rats as we previously reported may also contribute to the lower systemic exposure of GS after oral administration of GJ (Li et al., 2020). However, the other possible mechanisms underlying such alteration remained uninvestigated owing to limited understanding about the GS ADME. Additionally, the pharmacokinetic profiles of SV and SVA in NASH rats were not influenced by the coadministration of GJ. Consistently, the hepatic P-gp expression was not induced by the coadministration of GJ in NASH rats, which was not in accordance with the alterations in healthy rats. There were two potential explanations for such difference: one is that the systemic exposure of GS in NASH rats was lower than that in healthy rats after coadministration of SV and GJ, which may result in less potent up-regulation on the expression of P-gp; the other is that the hepatic protein level of P-gp in NASH rats was much higher than that in the healthy rats (Li et al., 2020); therefore, the treatment of GJ was less likely to further up-regulate the P-gp expression. Besides, it was noticed that in NASH rats, the down-regulation on the expression of Cyp2c11 in healthy rats mediated by SV was not observed, which might be due to the lower systemic exposures of SV and SVA in NASH rats compared to that in healthy rats.

Interestingly, severe liver injuries including inflammation, ballooning, and fibrosis were observed in both healthy and NASH rats which received two-week SV and GJ coadministration at 90 days after discontinuation of the herb/drug treatments. By comparison, such severe hepatic lesions were not observed in the healthy/NASH rats which received two-week treatment of GJ, demonstrating that the interaction between SV and GJ induced irreversible long-term liver injury in both healthy and NASH rats. Considering that inter- and intra-molecular crosslinking could happen among GP-protein adducts to form polymers including fibers (Neri-Numa et al., 2017), such long-term damage, especially the severe fibrosis, in the livers of healthy and NASH rats which received SV and GJ coadministration was proposed to result from the GP-protein adduct. The more severe fibrosis observed in the liver of healthy rats which received SV and GJ coadministration compared to that in NASH rats was consistent with its higher hepatic content of the GP-protein adducts. Two-week coadministration of SV and GJ exerted instant hepatoprotective effect in NASH rats via reducing the expression levels of iNOS/COX-1 and hepatic inflammation infiltration, however, resulting in long-term liver injury after further holding for 90 days. Such discrepancy between the short-term and long-term observations could be due to that the undetectable GP-protein adducts in the liver after the two-week treatment further crosslinked leading to severe liver injury at 90 days. Although our current investigations on the interactions between SV and GJ were originated from the observations in stroke rehabilitation patients, liver injury induced by such interactions could occur to patients with many other diseases. For patients who suffered diseases like jaundice, vexation, insomnia, hepatitis, and gastric ulcers, it is highly recommended to avoid concomitant usage of GJ with SV. When co-treatment of SV and GJ is unavoidably needed in clinical practice, the liver status of patients should be checked before the start of treatment. During such combination treatment, liver functions of the patients should be carefully monitored via detecting the serum ALT and AST levels, especially for those with an initially healthy liver condition.

## Conclusion

Our findings documented the disease–drug–herb interactions among NASH, SV, and GJ in rats. In healthy rats, both SV and GJ treatments were safe, while their coadministration induced significant liver injury, which might result from the SV-mediated higher systemic exposure of GS and the subsequent aggravated formation of the hepatotoxic GP-protein adduct. Such liver injury progressed to more severe liver damage after 90 days with discontinuation of herb/drug treatment. In NASH rats, both SV and GJ showed hepatoprotective and lipid-lowering activities, and their co-use exerted similar hepatoprotective effects and enhanced lipid-lowering activity. Although the coadministration of SV and GJ also induced liver damage in NASH rats after 90-day discontinuation of herb/drug treatment, such damage was milder than that in healthy rats. Therefore, NASH condition protected the rats from liver injury in short term and reduced the liver damage in long term that resulted from the interactions between SV and GJ. In general, SV and GJ are not recommended to be concomitantly used in both healthy and NASH conditions.

## Data Availability

The raw data supporting the conclusions of this article will be made available by the authors, without undue reservation.
