# Supplementary material for: Disease Status–Dependent Drug–Herb Interactions: NASH Lowered the Risk of Hepatotoxicity in Rats Coadministered With Simvastatin and Gardenia jasminoides J. Ellis
Source: Front Pharmacol. 2021 Apr 23;12:622040. doi: 10.3389/fphar.2021.622040 (PMC8103205; doi:10.3389/fphar.2021.622040)
Supplement: Supplementary file 1 [file DataSheet1.docx]

##### **Quantifications of GS and GP in the concentrated GJ granules**

***Methods***

The concentrated GJ granules were dissolved in 20% methanol (in water) to reach a final concentration of 2 mg/mL followed by sonication for 20 min. After centrifugation of the resulted mixture at 15,000 rpm for 10 min, 10 µL of the supernatant was injected into HPLC-UV for content analyses of GS and GP. Stock standard solution contains GS (1 mg/mL) and GP (1 mg/mL) was prepared by dissolving the standard compounds in 20% methanol. The stock standard solutions were diluted using 20% methanol to prepare a series of working solutions with final concentrations of 0.001, 0.005, 0.01, 0.02, 0.05, 0.1 and 0.5 mg/mL and injected for the analyses via HPLC-UV to obtain the standard curves of GS and GP.

The HPLC-UV system consisted of a Waters 600 controller (pump) coupled with a Waters 717 auto sampler and a Waters 2487 dual wavelength detector. GS and GP were separated using Sunfire C_18_ (5 µm, 3 mm×150 mm) column (Waters, MA, USA) with isocratic elution containing 50% acetonitrile and 50% 0.1% formic acid (in water) at flow rate of 1.0 mL/min. Both GS and GP were detected under the maximum wavelength of 238 nm, and their contents in the GJ granules were calculated based on their corresponding standard curves.

***Results***

The representative HPLC-UV spectrums of GS and GP in the standard solution and GJ granules sample at 238 nm were shown in **Figure S1**. Based on the standard curves, the concentration of GS in the solution of concentrated GJ granules (2 mg/mL) was determined to be 0.21 mg/mL, whereas the concentration of GP was below its lower limit of detection (0.001 mg/mL). Therefore, it was determined that the concentrated GJ granules contain 10.50% (w/w) GS with undetectable GP (<0.05%, w/w).

**Figure S1** Representative HPLC-UV spectrums of GS and GP in standard solution (0.01 mg/mL of both compounds) and GJ granules solution (2 mg/mL) at 238 nm.

**Cytotoxicities of GS (40 μM) in presence and absence of verapamil (100 μM) or SV (10 μM) in MDCKII-MDR1 cells**

***Methods***

MDCKII-MDR1 cells were cultured using Dulbecco's Modified Eagle Medium (DMEM) supplemented with 10% fetal bovine serum (FBS), penicillin (100 U/mL) and streptomycin (100 μg/mL) in cell incubator (37˚C, 5% CO_2_ with 90% relative humidity). For cytotoxicity studies, the cells were seeded onto 96-well plates at the density of 5×10^3^ cells/well and cultured for 24 h in the cell incubator. After that, the cells of each well were incubated with 200 μL of blank HBSS, HBSS contains GS (40 μM), HBSS contains verapamil (100 μM) and GS (40 μM), or HBSS contains SV (10 μM) and GS (40 μM) (triplicates for each test) in the cell incubator for 4 h. After the incubation, the medium was replaced by 200 μL of methyl thiazolyl tetrazolium (MTT) reagent (5 mg/mL dissolved in PBS) followed by incubation for another 2 h in the cell incubator. At the end of the incubation, after removing the solution in each well, 200 μL of DMSO was added to dissolve the purple formazan product for the further detection of optical density (OD) value at 490 nm.

Cytotoxicity of GS/SV/verapamil was indicated by the relative cell viability (%) calculated based on following equation.

Relative cell viability (%) = $\frac{{OD}_{490, sample} - {OD}_{490, blank}}{{OD}_{490, control} - {OD}_{490, blank}}\times100\%$

OD_490,sample_ is the average OD value at 490 nm of the compound treated MDCKII-MDR1 cells; OD_490,control_ is the average OD value at 490 nm of the blank HBSS treated MDCKII-MDR1 cells; OD_490,blank_ is the average OD value at 490 nm of the well contains blank HBSS.

***Results***

As shown in **Figure S2**, the variablities of MDCKII-MDR1 cells after incubating with GS (40 µM), GS (40 µM) and VP (100 µM), and GS (40 µM) and SV (10 µM) for 4 h were all more than 80%, indicating not significant cytotoxicity.

Figure S2 Cytotoxicities of GS (40 µM), GS (40 µM) and verapamil (VP, 100 µM), and GS (40 µM) and SV (10 µM) in MDCKII-MDR1 cells.
